# Systemic Risk Analysis of Multi-Layer Financial Network System Based on Multiple Interconnections between Banks, Firms, and Assets

**DOI:** 10.3390/e24091252

**Published:** 2022-09-06

**Authors:** Qianqian Gao

**Affiliations:** School of Financial Technology, Shanghai Lixin University of Accounting and Finance, Shanghai 201209, China; gaoqianqian@lixin.edu.cn

**Keywords:** multi-agent, multi-layer financial network, systemic risk

## Abstract

Global financial systems are increasingly interconnected, and risks can spread more easily, potentially causing systemic risks. Research on systemic risk based on multi-layer financial networks is relatively scarce, and studies usually focus on only one type of risk. This paper develops a model of the multi-layer financial network system based on three types of links: firm-bank credit, asset-bank portfolio, and interbank lending, which simulates systemic risk under three risk sources: firm credit default, asset depreciation, and bank bankruptcy. The impact of the multi-layer financial network structure, default risk threshold, and bank asset allocation strategy is further explored. It has been shown that the larger the risk shock, the greater the systemic risk under different risk sources, and the risk propagation cycle tends to rise and then decline. As centralized nodes in the multi-layer financial network system, bank nodes may play both blocking and propagation roles under different risk sources. Furthermore, the multi-layer financial network system is most susceptible to bank bankruptcy risk, followed by firm credit default risk. Further research indicates that increasing the average degree of firms in the bank–firm credit network, the density of the bank-asset portfolio network, and the bank capital adequacy ratio all contribute to reducing systemic risk under the three risk sources. Additionally, the more assets a bank holds in a single market, the more vulnerable it is to the risks associated with that market.

## 1. Introduction

Since 2008, research on systemic risk has gained increasing attention. Due to the complexity of the financial system, complex network theory has been utilized to study financial risk to fully understand the complex connections among the components of the financial system, as well as to investigate the mechanisms involved in the generation and propagation of financial risk within the whole complex system. Under the impact of COVID-19, and in the context of current deeper economic globalization, the links among agents in the financial system have become more closely linked, and the types of links have also become more diverse, providing more channels for the spread of financial risks. In this paper, three agents in the financial system are fully considered: banks, assets, and firms. It constructs a multi-layer financial network system model based on bank–firm credit linkages, bank-asset portfolio linkages, and interbank lending linkages, and investigates the systemic risk of the multi-layer financial network system through numerical simulation.

Previous research on systemic risk has focused on single-layer financial networks, such as the interbank lending network, bank-asset portfolio network, and bank–firm credit network. Financial risk can be spread directly between banks through interbank lending. Thus, many studies begin by examining how interbank lending linkages affect financial risk [1,2,3,4,5,6,7,8,9,10,11], most of which demonstrate that interbank lending linkages contribute to both the sharing of risk and the spreading of risk. Since different financial network systems typically have distinct structural characteristics, some studies have empirically analyzed the structural characteristics of interbank networks by utilizing actual interbank data. There have also been studies where the systemic risk has been simulated and analyzed under different network structures, assuming the interbank networks are structured differently. Several empirical studies have demonstrated that interbank networks exhibit characteristics of small-world networks or scale-free networks [12,13,14,15,16,17,18,19]. Further, Lenzu and Tedeschi [20], as well as Li and He [21], compared the systemic risk of financial systems based on different network structures through numerical simulations.

Aside from the studies based on interbank lending networks, there are also studies based on single-layer bank-asset portfolio networks and single-layer bank–firm credit networks. Huang et al. [22] presented a bilateral banking network model of banks and assets and proposed a cascading failure model to describe the risk propagation process. The study based on data from US commercial banks in 2007 found that commercial real estate assets, rather than residential real estate assets, were responsible for the failure of more than 350 US commercial banks from 2008 to 2011. A model based on Huang et al. [22] was developed by Levy-Carciente et al. [23], which was used to investigate the sensitivity of different asset classes to financial systemic risks in response to external shocks using data from the Venezuelan banking system from 2005 to 2013. Caccioli et al. [24] examined the impact of portfolio overlap on the stability of the bank-asset bilateral network. There exists a critical value of leverage ratio below which the financial network system is always stable. However, above the critical value, the region of instability increases with the leverage ratio. In addition, the dynamic deleveraging during times of crisis aggravates financial instability. Furthermore, Paulin et al. [25] focused on the impact of fund-asset networks on systemic risk, in particular, the impact of alternative network structures of overlapping asset portfolios. There is evidence that the rate of financial risk contagion is not a monotonic function of portfolio diversification when asset portfolios have a high degree of overlap. In some cases, increasing portfolio overlap is beneficial to financial system stability.

In contrast, Di Guilmi et al. [26] used statistical physics methods to characterize the behavior of firms and banks, as well as the dynamic evolution of the bank–firm credit network. In this study, there are no restrictions on the number of agents or the configuration of the network, and the network model is more flexible. Based on the debt ranking method (DebtRank), Li et al. [27] considered the factors of credit easing policy and constructed a model for bank–firm systemic risk measurement. They examined the systemic risk contribution and risk contagion effect of Chinese banks and firms based on the 2018 bank–firm lending data in China. According to the study, there are a few systemically important banks and firms whose systemic risk contribution is high. The degree of easing of credit policy also has a positive impact on the stability of the bank–firm credit system.

Due to the widespread application of complex network theory in financial research, some scholars have begun to conduct systemic risk research based on multi-layer financial networks in addition to single-layer financial networks. Due to their similarities, “multi-layer networks” and “multiplex networks” are often confused or even considered the same in some studies. The multi-layer network is generally more extensive than the multiplex network, which includes multiplex networks. A multiplex network may contain different types of connections, but the agent is the same type at each layer, according to Berndsen et al. [28] and Kivelä et al. [29]. In the beginning, some studies primarily used a large amount of actual data from the banking system, decomposed the single-layer network based on the bank lending cycle, asset types in banks, etc., to construct a multiplex financial network and assess systemic risk under different network layers. For example, Langfield et al. [30] and Poledna et al. [31] constructed multiplex financial networks using actual banking data. Based on the dataset of UK banks, Langfield et al. [30] built an interbank exposure network and an interbank funding network. According to their findings, the two networks have different structural characteristics, and their contagion processes differ in the interbank network system. Using actual data from the Mexican banking system between 2007 and 2013, Poledna et al. [31] constructed a four-layer Mexican interbank network and quantitatively analyzed each layer’s contribution to systemic risk. The study demonstrates that considering only a single layer of the network would significantly underestimate systemic risk and that the sum of all the systemic risks of all network layers would underestimate the overall risk.

Not limited to the multiplex case, the multi-layer network does not only contain different network layers, but also consists of multiple agents (including different types), and the agents are interconnected. There are some studies that construct a multi-layer financial network based on the connections between two agents in the financial system. Usually, numerical simulations are used to examine the systemic risk of the multi-layer financial network system. For example, both Caccioli et al. [32] and Jiang and Fan [33] constructed a two-layer financial network system based on the interbank network and the bank-asset portfolio network. Following the research of Caccioli et al. on single-layer bilateral bank-asset networks [24], Caccioli et al. [32] examined systemic risk and its contagion through two channels: interbank counterparty risk and bank-asset overlapping portfolios. Study results show that when the two risk contagion channels are combined, risk contagion is significantly increased and systemic risk is also elevated. Based on the basic model of Caccioli et al. [32], Jiang and Fan [33] further examined the investment risk of banks and assessed the impact of bank investment on the stability of the multi-layer financial network system. The study shows that banks contribute to their stability by selling depreciating assets, and investment risk is an uncertainty that affects the stability of banks. In their work, Lux [34], Silva et al. [35], and Gao et al. [36] constructed the multi-layer financial network system based on interbank and bank–firm networks. Based on De Masi and Gallegati’s research on the actual data sets of Italy and Japan [37], Lux [34] proposed a generation algorithm for the bilateral bank–firm network and combined it with the interbank network to study risk contagion. The bank–firm counterparty risk channel is found to be more important for the contagion of defaults. Using actual data from the Brazilian banking industry and firms, Silva et al. [35] constructed interbank and bank–firm networks to assess the contribution of each sector to the systemic risk in banking. It has been found that state-owned banks are most vulnerable to risky shocks. In addition, the network structure of the financial system plays an important role in the contagion of risk. Gao et al. [36] studied the systemic risk of the multi-layer financial network system under macroeconomic fluctuations and found that firms with medium and high leverage and small asset sizes, as well as banks with smaller asset sizes and fewer bank–firm credit links, are more likely to default. On the assumption that banks do not recover loans from firms, Chen et al. [38] constructed a two-layer credit risk contagion network model between banks and firm counterparties. In multi-layer bank–firm credit networks, they assessed the impact of relevant factors in inter-firm credit-related networks and inter-bank credit-related networks on counterparty credit risk.

There is no doubt that the current research on systemic risk based on complex networks has produced a wealth of research findings. It is also noteworthy that there are more studies on systemic risk that are based on single-layer financial networks, whereas there are fewer similar studies based on multi-layer financial networks, particularly those that consider multiple agents and their interconnections. Considering the diversity and complexity of current financial agents as well as the interconnections among them, further research is necessary on the complex multi-layer financial network system. In addition, the few studies that have examined multi-layer financial network systems usually consider the systemic risk of such systems under different propagation channels of risk when shocks occur. However, the impact of different types of risks and characteristics of different agents is not considered.

Based on the bank–firm credit linkages, the bank-asset portfolio linkages, and the interbank lending linkages, this paper develops a multi-layer financial network system model. According to this model, this paper examines the systemic risk of the multi-layer financial network system under three different risk sources, including firm credit default, asset depreciation, and bank bankruptcy. Several relevant parameters are then selected from three aspects, namely, the structure of the multi-layer financial network, the threshold value of default risk, and bank asset allocation strategy, to simulate their effects on the systemic risk of the multi-layer financial network system and make some suggestions for banks to avoid systemic risk.

The paper is strongly motivated by Lux [34]. Lux [34] proposes a generation algorithm for the bank–firm credit network and examines the impact of shocking different types of firms one-by-one using a two-layer financial network system model. Based on Lux’s study, this paper presents a more realistic complex financial system with multiple sources of risk by building a multi-layer financial network system model that incorporates multiple agents and their interconnections; it has also adopted some new research perspectives and obtained some new research findings. The contribution of this paper may be as follows: First, this paper proposes a method to build a bank-asset portfolio network based on network density and asset size, inspired by Lux’s generation algorithm for single-layer networks [34]. Furthermore, the paper considers three different agents, namely, banks, assets, and firms, and is inspired by research conducted by Levy-Carciente et al. [23], Caccioli et al. [32], and Lux [34], to portray the complex financial system more realistically, a multi-layer financial network system is formed with bank–firm credit, interbank lending, and bank-asset portfolio links with banks serving as the central nodes. As a result, research on systemic risk under multi-layered financial networks will be enriched. Moreover, this paper proposes a new research perspective and methodology. As an alternative to focusing on individual risks when studying systemic risk in multi-layer financial networks, this paper proposes a “shock event” involving three different risk sources: firm credit default, asset depreciation, and bank bankruptcy, focusing on the different types of risks and the impact of multiple key factors on systemic risk. In addition, combining the characteristics of multi-layer financial networks, the paper also considers bank losses in different financial markets on top of the traditional bank default probability and risk propagation cycle to comprehensively assess the systemic risk of the multi-layer financial network system. In this paper, recommendations are provided for banks to cope with systemic risks. The research results and conclusions presented in this paper may be helpful for financial institutions and regulators.

## 2. The Model

In this paper, three different agents are considered in the multi-layer financial network system model, namely banks, assets, and firms, which are interconnected to produce a complex multi-layer financial network system with banks as the center. In this multi-layer financial network system, firms seek loans from banks based on their production and operating needs, thus, forming a credit link between banks and firms from which a bank–firm credit network can be developed. A bank can also select to invest in different types of assets, creating an investment link between banks and assets, based on which a bank-asset portfolio network can be created. There are also interbank lending behaviors, and based on these interbank lending connections, an interbank lending network can be formed. Interbank lending is composed of nodes of the same type, which can be described as a unilateral network; the other two networks are comprised of nodes of different types, which can be described as bilateral networks. It is the superposition of the three networks that constitute the multi-layer financial network system presented in this paper.

### 2.1. Bank–Firm Credit Network

As a result of the credit relationship between banks and firms, firms can obtain the funds that they need to maintain their production and operations. In addition, banks can earn interest from lending money. The credit relationship between banks and firms can be expressed as a bilateral network, i.e., the network consists of two types of nodes: banks and firms. In addition, firm credit default is an important source of risk for banks. Bank–firm credit networks have shown that the number of bank nodes is much greater than that of firm nodes, and the credit linkages between banks and firms are unevenly distributed (large banks have credit links with many firms, while borrowers of small banks generally have fewer creditors).

By assuming that the balance sheet size of banks and firms follows the same Pareto distribution and that the connectivity of banks and firms is related to their size according to the scale-free network connectivity law, Lux [34] constructs a stochastic bank-asset bilateral network between all nodes based on stylized facts reported from actual bank-credit data in Italy and Japan. Lux’s algorithm generates a stochastic heterogeneous bank–firm bilateral network that matches the actual bank–firm credit data [34], and it does not require complex or detailed bank credit data, which provides great convenience for research. 

By setting the average connection degree among bank–firm nodes and calculating the connection degree of each node based on the asset size of the bank and the loan size of the firm, this paper uses the generation algorithm proposed by Lux [34] to construct a bank–firm credit network.

In this paper, the average connection degree of banks in the bank–firm credit network is $\lambda_{b}$. The connection degree of a bank is dependent on its relative asset size in the banking system; hence, the connection degree of bank i is as follows:

(1) $$\lambda_{i}=\lambda_{b}\frac{A_{i}}{{\bar{A}}_{i}}$$

where $A_{i}$ represents the total assets of bank i, and ${\bar{A}}_{i}$ represents the average total assets of all banks in the system. In this model, the size of the bank’s assets follows the Pareto distribution. Referring to Lux [34], the total assets $A_{i}$ of bank i are distributed as follows:

(2) $$f(A_{i})∼\frac{\alpha L^{\alpha }A_{i}^{-\alpha -1}}{1-{\left(\frac{L}{H}\right)}^{\alpha }}$$

where L and H are the total assets of the banks with the smallest and largest asset sizes in the bank–firm credit network, respectively, and α represents the Pareto distribution parameter.

In the bank–firm credit network, the sum of the banks’ degrees equals the sum of the firms’ degrees. Therefore, if the average connection degree of firms is $\lambda_{f}$, the number of firms in the bank–firm credit network is $N_{f}$, and the number of banks is $N_{b}$, then $\lambda_{f}=\lambda_{b}\frac{N_{b}}{N_{f}}$. In addition, the degree of the firm is also related to its borrowing size; hence, the connection degree of firm j can be expressed as follows:

(3) $$\lambda_{j}=\lambda_{f}\frac{D_{j}}{{\bar{D}}_{j}}$$

where $D_{j}$ denotes the borrowing size of firm j, and ${\bar{D}}_{j}$ denotes the average borrowing size of all firms in the system. The total amount of loans granted by banks is equal to the total amount of borrowings by firms in the bank–firm credit network. The average ratio of loans granted by banks to their total assets is θ, then there is $\theta \sum A_{i}=\sum D_{j}$, which can be simplified to obtain Equation (4) as follows:

(4) $${\bar{D}}_{j}=\theta {\bar{A}}_{i}\frac{N_{b}}{N_{f}}$$

Using the assumption of Lux [34], the borrowing size of firms follows the same Pareto distribution as that of banks, the minimum borrowing amount l and maximum borrowing amount h of firms can be expressed as follows:

(5) $$l=\theta L\frac{N_{b}}{N_{f}},\;h=\theta H\frac{N_{b}}{N_{f}}$$

Based on the above-mentioned connection degree of banks and firms, the scale-free network model generation algorithm proposed by Lux [34] and Goh et al. [39] is used in this paper to construct the bank–firm credit network. The degrees of bank and firm nodes are used to determine their initial available connections, and nodes are selected randomly from the bank and firm nodes, in turn, to establish credit connections and reduce the available connections of the corresponding nodes. The probability of a node being selected is related to its degree, and nodes with higher degrees have a greater chance of being selected. Upon exhaustion of all connections between nodes in the system, the credit linkage matrix $M^{bank-firm}$ between banks and firms is determined. The amount of the loan offered by bank i to firm j can be expressed as follows:

(6) $$D_{i,j}=\theta A_{i}\frac{D_{j}}{\sum_{k=1}^{N_{f}}D_{k}},(M_{i,j}^{bank-firm}=M_{i,k}^{bank-firm}=1)$$

where $M_{i,k}^{bank-firm}=1$ indicates that bank i has a credit link with firm k.

### 2.2. Bank-Asset Portfolio Network

Besides the bank–firm credit network, a bilateral network of bank-asset portfolios can be established based on the overlapping portfolios of different banks in the complex financial system. The price fluctuations of assets will affect the banks that invest in them in the bank-asset portfolio network. At the same time, banks can also transfer the risk to other banks through the sale of their investments, thus, forming an indirect channel for the transmission of risk. 

This paper proposes a method to construct a bank-asset portfolio network based on network density and asset size, inspired by Lux’s [34] single-layer network. It is assumed that $N_{b}$ banks cross-invest in $N_{a}$ types of assets in the bank-asset portfolio network. In this paper, we adjust the overlap between each bank’s portfolio by controlling the density ρ (ρ∈[0,1]) of the bank-asset portfolio network. The bank-asset portfolio network is fully connected when ρ=1, which means that the bank will invest in all types of assets. In the bank-asset portfolio network, the average degree of each type of asset can be expressed as follows:

(7) $$\lambda_{a}=\rho N_{b}$$

The total amount of various assets invested by bank i is as follows:

(8) $$I_{i}=\sum_{m=1}^{N_{a}}u_{m}I_{i,m}$$

where $I_{i,m}$ denotes the share of bank i investment in asset m, $u_{m}$ denotes the price of asset m. If the average proportion of various types of assets invested by a bank to its total assets is η, then the total value of the assets invested by all banks is $\eta \sum_{i=1}^{N_{b}}A_{i}$. Since the portfolios of banks are highly similar (high-quality assets are more likely to attract investors), the total value of asset m can be expressed as follows:

(9) $$NW_{m}=\frac{\lambda_{m}\eta }{\lambda_{a}N_{a}}\sum_{i=1}^{N_{b}}A_{i}$$

The total amount of investment in asset m by bank i can be expressed as follows:

(10) $$u_{m}I_{i,m}=I_{i}\frac{NW_{m}}{\sum_{n=1}^{N_{a}}NW_{n}},(M_{i,m}^{bank-asset}=M_{i,n}^{bank-asset}=1)$$

where $M_{i,m}^{bank-asset}=1$ indicates the bank i investment asset m. Finally, this paper obtains the bank-asset portfolio network by applying Equations (7)–(10).

### 2.3. Interbank Lending Network

In the banking system, interbank lending has been extensively studied as a direct channel of risk transmission. The interbank lending linkage matrix is constructed using a probability function related to the size of the bank’s assets, as described in Lux [34]. The probability of an interbank lending linkage between bank i and bank k is presented in this paper as follows:

(11) $$p_{ik}=P(A_{i},A_{k})=d{\left(\frac{A_{i}}{A_{\max}}\right)}^{\alpha_{1}}{\left(\frac{A_{k}}{A_{\max}}\right)}^{\alpha_{2}}$$

where $A_{i}$ and $A_{k}$ denote the total assets of bank i and bank k, respectively, and $A_{\max}$ represents the total assets of the largest bank node in the system. According to the above settings in the bank–firm credit network and bank-asset portfolio network, the ratio of the bank’s interbank assets to its total assets can be expressed as 1−θ−η. Therefore, the amount of the interbank lending from bank i to bank k can be expressed as

(12) $$IL_{ik}=\frac{(1-\theta -\eta )A_{i}p_{ik}A_{k}}{\sum_{h=1}^{N_{b}}p_{ih}A_{h}},(M_{i,k}^{bank-bank}=M_{i,h}^{bank-bank}=1)$$

where $M_{i,h}^{bank-bank}=1$ indicates that bank i has interbank lending relationships with bank h. In this paper, the interbank lending network can be estimated utilizing Equations (11) and (12).

### 2.4. Dynamic Evolution of the Multi-Layer Financial Network System

In this paper, three risk propagation channels are established through the linkages between banks, assets, and firms, namely, the direct bank–bank propagation channel and two indirect propagation channels, the bank–firm–bank, and the bank–asset–bank, that allow risk to propagate among the three single-layer networks, thereby developing the dynamic multi-layer financial network system model. The balance sheet of the bank in the multi-layer financial network system of this paper is shown in Table 1.

The total assets of bank i in the multi-layer financial network system consist of four parts, namely, operating loans to firms $A_{i}^{bank-firm}=\sum_{j=1}^{N_{f}}D_{i,j}$, investment portfolios (In this model, all bank assets except credit, interbank loans, and cash assets have been simplified into the bank’s investment assets, including investment real estate, long-term equity investments, derivative financial assets, bond assets, all real assets, and other assets. The majority of these assets are financial investment assets, and since all of these assets can be sold, they are all classified as bank investment assets) $A_{i}^{bank-asset}=\sum_{m=1}^{N_{a}}NW_{i,m}$, interbank loans to other banks $A_{i}^{bank-bank}=\sum_{k=1}^{N_{b}}IL_{i,k}$, and cash assets $A_{i}^{cash}$. On the other hand, the total liabilities of bank i are divided into three parts, which include the bank’s net equity $L_{i}^{bank-equity}$, depositor savings $L_{i}^{bank-deposit}$, and interbank borrowing from other banks $L_{i}^{bank-bank}=\sum_{k=1}^{N_{b}}IL_{k,i}$. The capital adequacy ratio of bank i is the ratio of net equity to weighted risk assets. According to the model in this paper, cash is a risk-free asset, and the risk weight of the remaining assets is 100%. Therefore, the capital adequacy ratio of bank i at time t can be expressed as follows:

(13) $$\omega_{i}(t)=\frac{L_{i}^{bank-equity}}{A_{i}^{bank-firm}+A_{i}^{bank-asset}+A_{i}^{bank-bank}}$$

A shock event containing three different risk sources (firm credit default, asset depreciation, and bank bankruptcy) is defined in the multi-layer financial network model in this paper, accounting for the different characteristics of firms, assets, and banks.

① At time t, the loan acquisition rate of firm j can be expressed as $\psi_{j}(t)$. If $\psi_{j}(t)<\psi_{0}$, firm j will default, and $\psi_{0}$ denotes the minimum loan acquisition rate of the firm. When a firm defaults, its creditor bank i will suffer asset losses and, thus, the balance sheet of bank i at time t is updated according to Equation (14).

(14) $$\left\{ \begin{array}{l} A_{i}^{bank-firm}(t)=A_{i}^{bank-firm}(t-1)-V_{ij}^{bank-firm}(t-1)\\ L_{i}^{bank-equity}(t)=L_{i}^{bank-equity}(t-1)-V_{ij}^{bank-firm}(t-1)\\ V_{ij}^{bank-firm}(t)=0 \end{array}\right.$$

where $V_{ij}^{bank-firm}(t-1)$ is the loan amount lent by bank i to firm j.

② In addition to firm credit defaults, banks are also exposed to asset depreciation risks. The balance sheet of bank i, which invests in the depreciating asset m, is updated according to Equation (15) if asset m depreciates at time t.

(15) $$\left\{ \begin{array}{l} A_{i}^{bank-asset}(t)=A_{i}^{bank-asset}(t-1)-\sum \Delta V_{im}^{bank-asset}(t)\\ L_{i}^{bank-equity}(t)=L_{i}^{bank-equity}(t-1)-\sum \Delta V_{im}^{bank-asset}(t)\\ \Delta V_{im}^{bank-asset}(t)=V_{im}^{bank-asset}(t)-V_{im}^{bank-asset}(t-1) \end{array}\right.$$

where $\Delta V_{im}^{bank-asset}(t)$ represents the depreciation loss incurred by bank i on its investment in asset m.

③ In the model of this paper, banks may fail due to both firm credit defaults and asset depreciation; meanwhile, they may also fail due to external factors that adversely affect firm credit and asset values. If the capital adequacy ratio of bank i at time t meets $\omega_{i}(t)\leq 0$, bank i fails and is liquidated. In the bankruptcy liquidation of the bank, its holdings will be sold first, and its loans will be recovered.

The sale of assets by banks will result in the depreciation of those assets, which will further spread risk within the banking system. This paper refers to the study of Caccioli et al. [24] and introduces a market impact function to measure the markdown sale effect of assets, as shown in Equation (16):

(16) $$g(x_{m})=e^{-\sigma x_{m}}$$

$x_{m}$ denotes the ratio between the assets m to be sold by the failed bank and the total assets m in the system, and σ denotes the sensitivity of an asset price to a sale at a reduced price. At time t, the price of asset m will be updated as follows:

(17) $$u_{m}(t)=u_{m}(t-1)g(x_{m},t)$$

As a result, the unbroken bank i holding asset m will trigger risk source ②, and its balance sheet will also be updated following Equation (15), where $V_{im}^{bank-asset}(t)=V_{im}^{bank-asset}(t-1)\frac{u_{m}(t)}{u_{m}(t-1)}$.

The bank’s recovery of firm loans may also lead to the default of its debt firm j. In such a case, the unbroken creditor bank i of firm j will trigger risk source ①, and its balance sheet will be updated following Equation (14).

It should be noted that if bank k fails and exits, it will repay its interbank borrowing after liquidating its assets, and the balance sheet of its creditor bank i will be updated as follows.

(18) $$\left\{ \begin{array}{l} A_{i}^{bank-bank}(t)=A_{i}^{bank-bank}(t-1)-V_{ik}^{bank-bank}(t-1)\\ L_{i}^{bank-equity}(t)=L_{i}^{bank-equity}(t-1)-\Delta V_{ik}^{bank-bank}(t)\\ \Delta V_{ik}^{bank-bank}(t)=-\frac{L_{k}^{bank-equity}(t)}{L_{k}^{bank-bank}(t-1)}V_{ik}^{bank-bank}(t-1)\\ V_{ik}^{bank-bank}(t)=0\\ A_{i}^{cash}(t)=V_{ik}^{bank-bank}(t-1)-\Delta V_{ik}^{bank-bank}(t) \end{array}\right.$$

where $V_{ik}^{bank-bank}$ denotes the amount of debt between bank i and bank k, $\Delta V_{ik}^{bank-bank}$ denotes the loss suffered by bank i ($L_{k}^{bank-equity}(t)<0$), and the remaining principal balance of the loan issued by bank i to the bankrupt bank k, less the loss, will be converted into risk-free assets.

Some key variables and parameters have been sorted out, which are in Table 2.

Based on the above behavior, the dynamic evolution process of the multi-layer financial network system model in this paper can be described as follows:

- The system is exposed to the risk of firm credit default, asset depreciation, and bank bankruptcy, respectively.
- Update the bank–firm credit matrix, the bank-asset portfolio matrix, and the interbank lending matrix, as well as calculate and update the balance sheets and current states of banks.
- If a bank fails and exits, it enters into bankruptcy liquidation, in which it will sell all the assets it has invested in, recover all firm loans and interbank loans, and repay its interbank borrowing debts.
- The other banks that invest in the same assets as the failed bank will suffer investment losses; firms whose loans are recovered will suffer loan losses and their loan acquisition rates will be reduced; creditor banks that have interbank lending linkages with the failed bank will also suffer lending losses.
- Calculate and update the balance sheet and state of each bank, the state of each firm, as well as the value of each asset; if the capital adequacy ratio of a new bank is less than or equal to zero, it will close and exit; if the loan acquisition rate of any firm is lower than the minimum loan acquisition rate of firms, it will fail and exit; the assets sold by failed banks will depreciate.
- Reproduce the above algorithm until there are no new defaults in the system.

**Figure 1:**
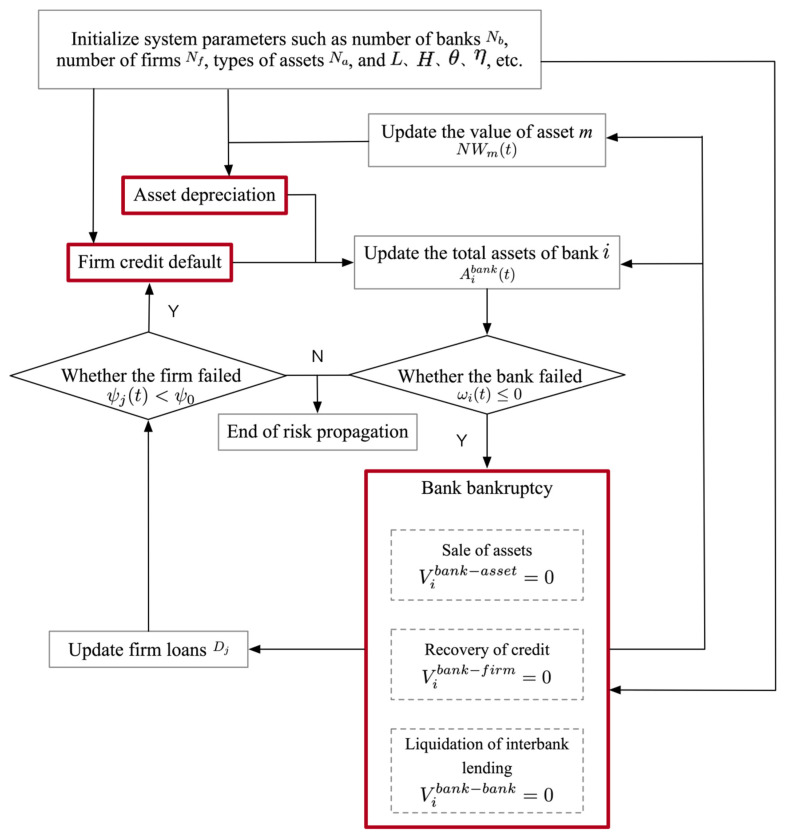
Dynamic evolution process of the multi-layer financial network system.

Figure 1 illustrates the dynamic evolution process of the multi-layer financial network system in this paper, and the parts marked in red indicate three different risk sources. For example, when a firm defaults on credit, if its creditor bank becomes insolvent due to losses, the liquidation of its creditor bank will cause other firms that borrowed from it to suffer losses in loan funds and may fail, which in turn will cause more banks to suffer credit losses. Meanwhile, the liquidation of the asset portfolio held by the insolvent bank will result in the depreciation of these assets. The banks holding these assets will also suffer losses.

### 2.5. Systemic Risk Measure

The multi-layer financial network system presented in this paper covers three different financial markets: credit, investment, and interbank lending. Banks serve as the central node in the network, providing direct or indirect connections among the agents through the three different financial markets, which also provide channels for the cross-market transmission of risk. To assess the systemic risk of the multi-layer financial network system, this study measures bank default probability, bank risk loss, and risk propagation cycle of the financial network system.

#### 2.5.1. Bank Default Probability

In a multi-layer financial network system with $N_{b}$ banks, we define CDP as the cumulative default probability and DDP as the direct default probability of banks caused by the risk source *r* as follows:

(19) $$\left\{ \begin{array}{l} CDP(r,t)=\frac{1}{n}\sum \frac{F_{b}(t)}{N_{b}}\\ DDP(r)=ADP(r,1) \end{array}\right.$$

where n represents the number of simulation experiments, and $F_{b}(t)$ represents the cumulative number of failed banks at time t. 

#### 2.5.2. Bank Risk Loss

We define the asset loss suffered by the banking system to be the difference between the total value of assets at time t and the initial total value of assets. Following n times of simulation experiments, the bank’s asset loss in the financial market c caused by the source of risk r and its proportion to the overall loss of all financial markets can be expressed as follows:

(20) $$\left\{ \begin{array}{l} Loss(r,c,t)=\sum A_{i}^{bank-c}(t)-A_{i}^{bank-c}(t_{0})\\ LR(r,c,t)=\frac{1}{n}\sum \frac{Loss(r,c,t)}{\sum Loss(r,c,t)} \end{array}\right.$$

#### 2.5.3. Risk Propagation Cycle

In each simulation experiment, there are no longer new failures in the multi-layer financial network system after time t, i.e., the system reaches stability. Upon simulation of n times, the risk propagation cycle (RPC) caused by the risk source r in the multi-layer financial network system can be expressed as follows:

(21) $$RPC(r)=\frac{1}{n}\sum t$$

## 3. Numerical Simulation and Result Analysis

With reference to the number of listed banks, the number of listed firms, and the balance sheet information of listed banks in China (According to the findings of the existing related studies and based on actual data from the Chinese economy, there were 54 listed banks in China by the end of 2020, and 4154 listed firms. Thus, there are 4000 firms and 50 banks in this study. In addition, according to the balance sheet data of listed banks in China, the average assets invested by banks exceed 15 types, and taking into account the number of firms and banks in this study, a total of 20 types of assets are set in this paper), the number of banks is set at $N_{b}=50$, the number of firms is set at $N_{f}=4000$, and the total amount of various asset types held by the banks is set at $N_{a}=20$ in this paper’s multi-layer financial network system model. Based on data from the People’s Bank of China (By 2020, the core tier 1 capital adequacy ratio of China’s banking industry was 10.72%, the ratio of bank loan balances to its total assets was approximately 55.8%, and the ratio of bank deposit balances to its total assets was approximately 68.3%. Initializing the model, the bank’s initial net equity is assumed to account for 10% of the bank’s liabilities, the bank’s savings account for 70%, and the bank’s interbank borrowings are assumed to account for 20%. Correspondingly, the bank’s interbank loans also account for 20% of its assets on the balance sheet. Based on the above actual data and the convenience of the study, the bank’s initial cash is set at zero, the average ratio of firm loans is set at 50%, and given the balance sheet balance, the average ratio of total amount invested by the bank in all types of assets is 30%), the basic model of this paper assumes that the average proportion of total firm loans to total bank assets is θ=50%, the average ratio of the total assets invested by banks to their total assets is set at η=30%, and the average ratio of interbank lending assets to total assets is set at 1−θ−η=20%. Initially, set the bank’s capital adequacy ratio at ω=10%.

Additionally, referring to Lux [34], the average connection degree of firms is set at $\lambda_{f}=2$ in the base model of this paper, therefore, the average connection degree of banks is $\lambda_{b}=\lambda_{f}\frac{N_{f}}{N_{b}}=160$, and the minimum loan acquisition rate of firms is $\psi_{0}=0.8$. The density of the bank-asset portfolio network is set at ρ=0.3. To eliminate the possibility of randomness in simulation experiments, this paper conducts a total of 1000 simulations for each parameter setting.

Listed in Table 3 are the initial values of the key parameters in the model and their range of values.

**Figure 2:**
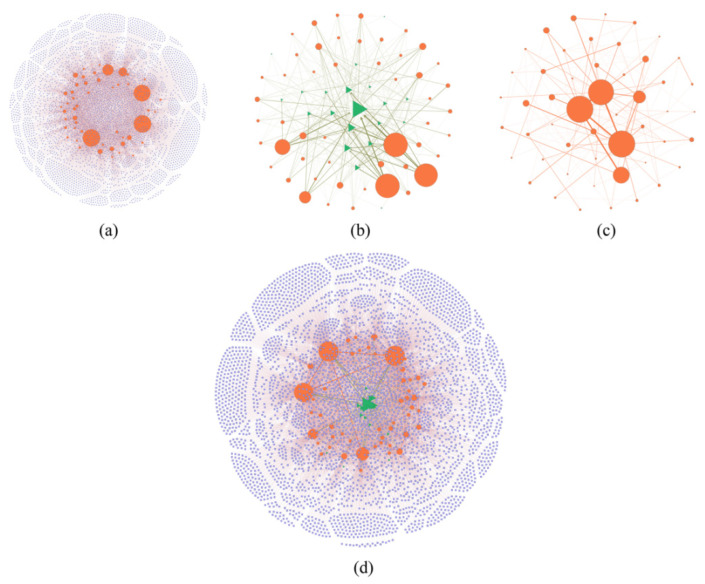
Illustration of single-layer and multi-layer financial networks. There are 4000 firm nodes, 50 bank nodes, and 20 asset nodes in the figure, where red dots represent banks, green dots represent assets, and blue dots represent firms; (**a**) represents bank–firm credit network; (**b**) represents bank-asset portfolio network; (**c**) represents interbank lending network; (**d**) represents a multi-layer financial network composed of banks, firms and assets, and their interconnections.

According to the parameter settings in the above model, the bank–firm credit network, bank-asset portfolio network, and interbank lending network are obtained as shown in Figure 2a–c, and the multi-layer financial network under the superposition of the three networks is shown in Figure 2d.

In this paper, the stress test method is used to carry out numerical simulations. Using the stress test approach, the model in this paper does not consider the impact of asset returns on the systemic risk of banks in the context of a multi-layer financial network, but rather examines how well banks are able to resist risk at a point in time when certain types of risks occur in a multi-layer financial network system due to external shocks. Firstly, this paper investigates the systemic risk of the multi-layer financial network system under the influence of three different risk sources separately. Randomly selecting a certain proportion of agents to cause them to incur default risk separately. Based on the influence of this risk, let it propagate the risk in the multi-layer financial network system following the evolutionary algorithm described in Section 2.4 of this paper. The systemic risk induced by different risk sources is simulated by gradually increasing their default proportions.

Based on the above basic research, moderate-sized risks are selected under three risk sources to study their impact on systemic risk by adjusting the structure of the multi-layer financial network, the default risk threshold, and the bank asset allocation strategy. Specifically, by adjusting both the average degree of firms and the density of the bank-asset network to influence the structure of the multi-layer financial network; by adjusting the capital adequacy ratios of banks and the minimum loan acquisition rate of firms to influence the threshold value of default risk; by adjusting the ratio of credit assets to total assets to influence the bank asset allocation strategy.

### 3.1. Results of the Base Model

**Figure 3:**
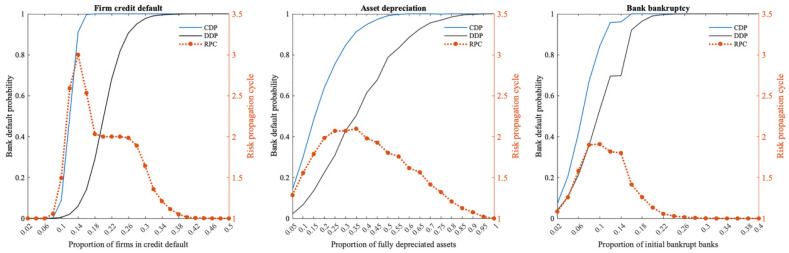
Systemic risk of the multi-layer financial network system under the impact of three risk sources. CDP indicates the cumulative default probability of banks; DDP indicates the direct default probability of banks; RPC indicates the risk propagation cycle.

Figure 3 illustrates the similar effects when different scales of firm credit default risk, asset depreciation risk, and bank bankruptcy risk occur within the multi-layer financial network system. All these studies demonstrate that the default probability of banks gradually increases over time as the size of risk shocks increases, and that the propagation cycle of risks within the multi-layer financial network system tends to increase and then decrease.

Clearly, in the multi-layer financial network system centered on banks, the greater the risk shock, the greater the loss the system suffers, and the more banks fail directly as a result. The risk sources in this model come from different agents in the financial market, but the risk can be transmitted through the complex interconnections between agents in the multi-layer financial network. Therefore, as risk shocks increase, the cumulative default probability of banks in the entire multi-layer financial network system increases.

Since the size of the different financial markets in the multi-layer financial network system differs, the sensitivity of the entire financial system to three different risk sources varies. According to the study, when 18% of firms in the multi-layer financial network system default on credit, 60% of assets depreciate completely, and 16% of banks fail, the whole banking system will collapse completely, and all banks will go bankrupt. This study shows that the multi-layer financial network system responds differently to different risk sources, i.e., it is most sensitive to bank bankruptcy, followed by firm credit default, and least sensitive to asset depreciation. In a multi-layer financial network system in which banks are the central nodes, the risk of bank bankruptcy will spread to multiple financial markets through bank–firm credit linkages, the investment portfolios od banks, and interbank lending relationships. By contrast, firm credit default risk and asset depreciation risk impact banks first, and after most of the risk has been absorbed by banks, the remaining risk spills over to other financial markets through banks. Therefore, compared to the other two risk sources, the proportion of bankrupt banks required to cause the entire banking system to collapse is the lowest. Due to the size of the market, firm credit accounts for a greater proportion of total bank assets than the portfolio held by banks, thus, by comparison, the banking system is more vulnerable to firm credit defaults. In the 2008 financial crisis, it was also demonstrated that when a bank fails, it will have a destructive effect on the entire financial system, and credit problems may trigger the failure of a bank before it fails.

Meanwhile, in terms of the risk propagation cycle triggered by shocks from different types of risk sources within the multi-layer financial network system, the greater the risk shock, the more risk spills outward through banks and, therefore, the longer the risk propagation cycle within the multi-layer financial network system. Nonetheless, as risk shocks continue to increase, more banks fail under direct shocks; as a result, indirect bank failures due to shocks in the multi-layer financial network system also decrease, and the risk propagation cycle will be shortened. As soon as the risk shock exceeds a certain size, all banks in the multi-layer financial network system fail under the direct shock, i.e., the risk propagation cycle is only one.

**Figure 4:**
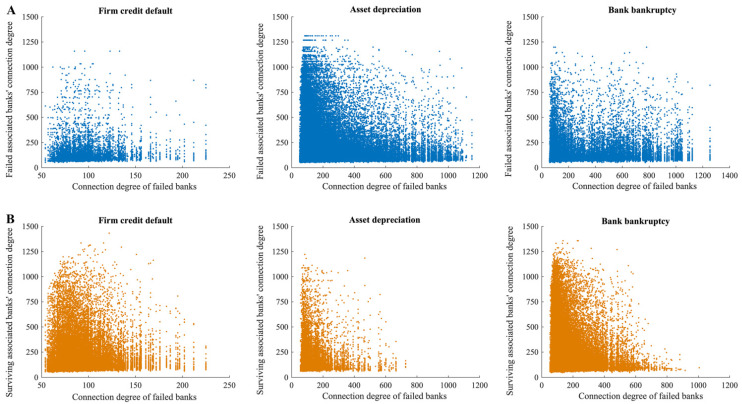
The connection degree of failed banks and their associated banks. ((**A**): The connection degree of failed banks and their associated failed banks; (**B**): The connection degree of failed banks and their associated surviving banks). Associated banks are those banks that have common borrowing firms, bank. There is a 12% probability of firm credit default, a 15% probability of asset depreciation, and a 6% probability of bank bankruptcy; therefore, the cumulative default probability caused by these three risk sources is roughly equal, approximately 50%.

From the perspective of the whole multi-layer financial network system, the bank node is the central node of the multi-layer financial network system, and the risks resulting from a single financial market risk source will be spread to other financial markets through the bank, resulting in a greater impact. From the perspective of the banking system, each bank plays a different role due to its own capital strength and position in the network. Banks can absorb risk losses caused by different risks and play a role in blocking risks on the one hand. Meanwhile, if a bank fails because of excessive losses, its liquidation will result in more risk losses, thus, causing risk transmission. As can be seen from the subplot on the left of Figure 4A, when firm credit default risk occurs, most of the banks that are affected by it and fail are those with relatively low connection degrees. As these banks are relatively small and their credit assets are more concentrated, they are more vulnerable to the impact of firm credit defaults. It is also found that when these banks fail, the failure of associated banks caused by the liquidation behavior of the failed banks is also concentrated in the bank with a lower connection degree, while the bank with a higher connection degree is less likely to fail. Thus, in the process of firm credit default risk spreading outward through failed banks, banks with a lower connection degree play a risk propagation role, while banks with a higher connection degree play a risk-blocking role.

When asset depreciation risk occurs (the subplot in the middle of Figure 4A), then all types of banks are likely to fail, depending on whether they hold depreciating assets. Unlike the case of firm credit default risk, the number of failed banks is higher due to the higher probability of direct bank failures caused by asset depreciation and, thus, the risk spread outward through the liquidation behavior of failed banks is also higher. The number of other banks with which the failed banks are associated survives significantly less than the number of failures (Figure 4B), and all types of banks play a greater role in the propagation of risk. At the same time, if the connection degree of banks that fail due to asset depreciation is too large, the default probability of other banks with which they are connected will be greatly increased.

When bank bankruptcy risk occurs (the subplot on the right of Figure 4A), since interbank loans account for a small proportion of total bank assets, the risk is mainly transmitted to other banks through co-borrowers or common assets held, so the risk will be diluted in the process of transmission. Therefore, the banks that fail due to the influence of bankrupt banks are primarily small, and their connection degree in the multi-layer financial network system is also low. Larger banks with higher connection degrees affected by it are less likely to fail. Similar to the situation when the system suffers from asset depreciation risk if the bank that fails is a bank with a higher connection degree, the default probability of other banks associated with that bank will also be higher due to the higher direct risk loss resulting from its liquidation.

Bank nodes are generally centralized nodes in the multi-layer financial network system, and they play different roles under different risk sources, which may be both risk blocking and risk propagation. Under the influence of different risk sources, bank bankruptcy risk has the greatest impact on the whole multi-layer financial network system, especially when the bank bankruptcy occurs in the banks with a higher connection degree in the multi-layer network system, which occupy the central position in the whole multi-layer financial network system. Since this risk is relatively unlikely to occur in the actual financial system, the firm credit default risk and asset depreciation risk deserve greater attention. In a multi-layer financial network system, this may be attributed to the close connection between the agents, the stability of the agents, or the bank’s asset allocation strategy.

Based on the above basic simulation results, this paper selects the shock sizes that cause similar bank default probabilities under three risk sources, and then investigates their impact on the systemic risk of the multi-layer financial network system from three perspectives: multi-layer financial network structure, default risk threshold, and bank asset allocation strategy.

### 3.2. The Multi-Layer Financial Network Structure

There are differences in the average degree of firms, which in turn affects the structure of the bank–firm credit network; the density of the bank-asset portfolio network also directly influences the portfolio held by banks. Through the connection between agents, changes in the structure of these two single-layer networks will also affect the structure of the multi-layer financial network system and, thus, affect systemic risk. To examine the impact of changes in the structure of the multi-layer financial network system on its systemic risk, the average degree of firms in the bank–firm credit network and the density of the bank-asset portfolio network are selected.

**Figure 5:**
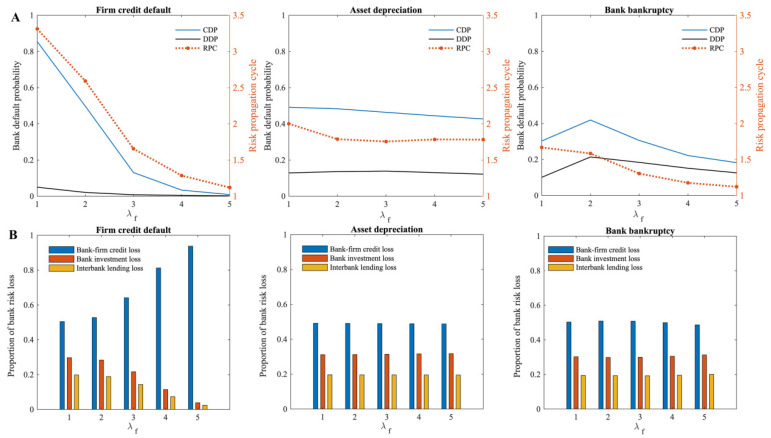
Systemic risk of the multi-layer financial network system under different $\lambda_{f}$. ((**A**): The default probability of banks and the risk propagation cycle under different $\lambda_{f}$; (**B**): The proportion of bank risk losses in different financial markets under different $\lambda_{f}$). CDP indicates the cumulative default probability of banks; DDP indicates the direct default probability of banks; RPC indicates the risk propagation cycle.

In this model, $\lambda_{f}$ represents the average degree of firms in the bank–firm credit network, i.e., the number of creditor banks. The effect of the average degree of firms ($\lambda_{f}$) on the systemic risk of the multi-layer financial network system is first examined. According to Figure 5, the greater the average degree of firms in the bank–firm credit network, the lower the systemic risk of the multi-layer financial network system when there is a risk of firm credit default or bank bankruptcy. There is little effect of the change in average firm degree in the bank–firm credit network when asset depreciation occurs on the systemic risk of the multi-layer financial network system.

The subplot on the left of Figure 5A shows that when the risk of firm credit default occurs, the higher the average degree of firms in the bank–firm credit network, the lower the cumulative default probability of banks in the multi-layer financial network system, and the shorter the risk propagation cycle. Additionally, as the average degree of firms in the bank–firm credit network increases, the greater the proportion of bank–firm credit losses in the multi-layer financial network system (Figure 5B), indicating that the systemic risk in multi-layer financial networks is increasingly concentrated in the bank–firm credit market. When bank bankruptcy risk occurs, the subplot on the right of Figure 5A shows that the cumulative default probability of banks shows a trend of slightly increasing and then decreasing, while the risk propagation cycle in the multi-layer financial network system decreases. The average degree of firms in the bank–firm credit network has a less pronounced impact on the systemic risk of the multi-layer financial network system when there is a bank bankruptcy risk than when there is a firm credit default risk. According to the subplot in the middle of Figure 5A, the average degree of firms in the bank–firm credit network has no significant impact on the systemic risk of the multi-layer financial network system when asset depreciation occurs, indicating a slight decrease in the systemic risk of the multi-layer financial network system as the average degree increases.

According to the analysis, the greater the average degree of firms in the bank–firm credit network, the greater the number of creditor banks of the firm, reducing the impact of firm credit default on individual creditor banks given the constant total amount of firm credit in the multi-layer financial network system. For its creditor banks, the larger the average degree of firms in the bank–firm credit network, the more the risk under that shock is spread out, and the less risk the banks have to bear. Therefore, increasing the average degree of firms in the bank–firm credit network may reduce the impact of firm credit defaults on the multi-layer financial network system’s systemic risk. Meanwhile, the greater the average degree of firms in the bank–firm credit network, the smaller the direct risk of firm credit defaults to banks, which means less risk of spillovers to other financial markets via banks, and the shorter the risk propagation cycle. Similarly, as the average degree of firms in the bank–firm credit network increases, the dependence of firms on individual creditor banks decreases. As a result, the less credit firms lose when there is a bank bankruptcy risk. It should be noted, however, that when the banks in the multi-layer financial network system go bankrupt, if the firm has fewer creditor banks ($\lambda_{f}\leq 2$), its risk diversification effect is not yet sufficient to prevent the firm from defaulting. This means that default of the creditor bank can still result in the credit loss of firms exceeding their affordability, resulting in an increase in systemic risk within the multi-layer financial network system. In cases where firms have enough creditor banks ($\lambda_{f}>2$), the default of a single creditor bank will not have a significant impact on the firm. Accordingly, when bank bankruptcy risk occurs, the systemic risk of the multi-layer financial network system tends to rise and then decrease with an increase in the average degree of firms in the bank–firm credit network. Correspondingly, as the average degree of firms in the bank–firm credit network increases, the risk that firms will fail as a result of credit losses decreases, making the risk of bank bankruptcy propagating to other banks weaker, and thereby the propagation cycle of this risk in the multi-layer financial network system is also shortened.

As the average degree of firms in the bank–firm credit network changes, there is no effect on the behavior of banks investing in various assets in the multi-layer financial network system. Consequently, when asset depreciation occurs, the direct default probability of banks in the multi-layer financial network system triggered by it changes little. Because of the increase in bank–firm credit links, spillover risk is further dispersed as the risk spreads through banks to the bank–firm credit market. Therefore, the indirect risk it causes diminishes. However, it only contributes to a modest reduction in the risk of bank–firm spillovers. Consequently, when the risk of asset depreciation occurs, the average degree of firms in the bank–firm credit network has less impact on the systemic risk of the multi-layer financial network system.

In summary, the greater the average degree of firms in the bank–firm credit network, the more diversified the bank–firm credit risk is in the multi-layer financial network system. Increasing the average degree of firms in the bank–firm credit network has a better effect on mitigating the systemic risk of the multi-layer financial network system when the risk of firm credit default or bank bankruptcy occurs. Since there is an asymmetry of information between borrowers and lenders in a market economy, banks should be cautious of firms providing false information to them to obtain loans. In a complex financial system, when banks evaluate whether to provide loans to firms, they should conduct a sufficient investigation and rigorous examination of the firms, and in addition to measuring the basic creditworthiness and operation of the firms, they should also consider the debt diversification of the firms to avoid excessive risk concentration.

**Figure 6:**
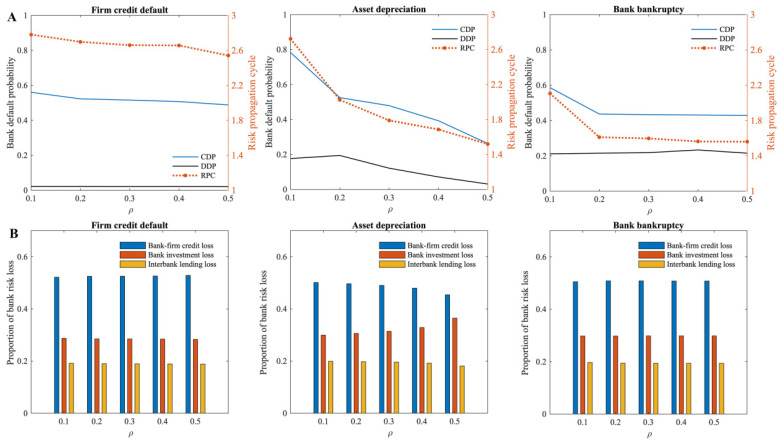
Systemic risk of the multi-layer financial network system under different ρ. ((**A**): The default probability of banks and the risk propagation cycle under different ρ; (**B**): The proportion of bank risk losses in different financial markets under different ρ). CDP indicates the cumulative default probability of banks; DDP indicates the direct default probability of banks; RPC indicates the risk propagation cycle.

Furthermore, we investigate the impact of bank-asset portfolio network density (ρ) on the systemic risk of the multi-layer financial network system. Generally, when asset depreciation risk occurs, as the density of the bank-asset portfolio network increases, it mitigates the systemic risk of the multi-layer financial network system, and the risk propagation cycle decreases sharply (the subplot in the middle of Figure 6A). Meanwhile, under this risk source, as the density of the bank-asset portfolio network increases, the proportion of bank investment losses increases, while the proportion of bank credit losses and interbank lending losses gradually decreases, indicating the concentration of risk on the bank investment market (the subplot in the middle of Figure 6B). There is no obvious effect of bank-asset portfolio network density on the systemic risk and the risk propagation cycle of the multi-layer financial network system when firm credit default risk and bank bankruptcy risk occur.

The analysis shows that the higher the density of the bank-asset portfolio network, the more investors there are in a single asset. Since the total amount of assets in the multi-layer financial network system remains unchanged when asset depreciation occurs, its impact on a single investor is smaller; at the same time, the investment risk of banks is also more dispersed, and single-asset depreciation has a smaller impact on them. Accordingly, as the network density of the bank-asset portfolio increases under this risk source, the cumulative default probability of banks in the multi-layer financial network system also gradually decreases. Furthermore, as the density of the bank-asset portfolio network increases, the risks faced by banks become dispersed, and the banks’ capacity to absorb risks increases, reducing risk spillover to other financial markets. Thus, when the risk of asset depreciation occurs, the risk propagation cycle in the multi-layer financial network system is shortened. Similarly, the asset classes in which banks invest increase as the density of the bank-asset portfolio network increases. It should be noted that if a bank experiences bankruptcy risk, its behavior of selling assets will have less impact on individual asset types. Therefore, the cumulative default probability of banks in the multi-layer financial network system under this risk source gradually decreases, and less risk is transferred to other banks through their assets, thus, reducing the risk propagation cycle.

Similar to the average degree of firms in the bank–firm credit network, adjustments in the density of the bank-asset portfolio network in the multi-layer financial network system do not affect the bank–firm credit. Therefore, when the risk of firm credit default occurs, the direct default probability of banks in the multi-layer financial network system does not change significantly. When the risk spreads through banks to the bank investment market, the higher the density of the bank-asset portfolio network, the greater its dispersion effect on spillover risks. As a result, the indirect risk it induces decreases. This improvement effect, however, is relatively small due to the size of the assets the banks hold. Overall, increasing the density of the bank-asset portfolio network slightly mitigates the systemic risk of the multi-layer financial network system when firm credit default occurs. To put it simply, in a multi-layer financial system, by increasing the density of the bank-asset portfolio network, a certain amount of systemic risk can be mitigated. Particularly, it plays a significant role in addressing asset depreciation risk. As a result, banks should increase the variety of their investment assets and reduce investment risk by diversifying their investments.

### 3.3. Default Risk Threshold

In the multi-layer financial network model of this paper, the bank’s capital adequacy ratio determines the bank’s net worth, i.e., the size of the risk buffer fund, which can affect the bank’s ability to tolerate risk. Additionally, the minimum loan acquisition rate of the firm reflects the firm’s dependency on external credit funding. The firm will be at risk of collapse if it is unable to obtain adequate credit, which in turn triggers additional credit defaults through the network of links in the multi-layer financial system. In this model, the capital adequacy ratio and the minimum loan acquisition rate of the firm represent the critical values of risk sensitivity, respectively, for banks and firms in the multi-layer financial network system. By adjusting the capital adequacy ratio of the bank and the minimum loan acquisition rate of the firm, this paper examines the effect of the threshold value of default risk on systemic risk of the multi-layer financial network system.

**Figure 7:**
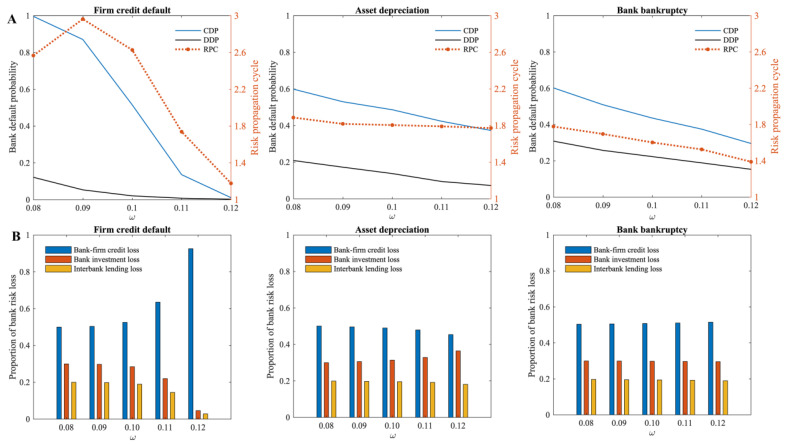
Systemic risk of the multi-layer financial network system under different ω. ((**A**): The default probability of banks and the risk propagation cycle under different ω; (**B**): The proportion of bank risk losses in different financial markets under different ω). CDP indicates the cumulative default probability of banks; DDP indicates the direct default probability of banks; RPC indicates the risk propagation cycle.

The higher the capital adequacy ratio of the bank (ω), the greater the risk buffer funds the bank has, and the stronger its ability to withstand risks. According to Figure 7A, when three different risks occur separately in the multi-layer financial network system, the cumulative default probability of banks decreases to various degrees as the capital adequacy ratio of the bank increases. Additionally, under its influence, the propagation cycle of risks in the multi-layer financial network system continues to shrink, indicating that the capital adequacy of banks also plays a significant role in preventing the propagation of risks in the multi-layer financial network system. When firm credit default risk occurs, the banks’ capital adequacy has the most significant effect on mitigating systemic risk. Moreover, as shown in Figure 7B, the proportion of risk losses in different markets indicates that when firm credit default risk occurs, the risk gradually accumulates in the bank–firm credit market as banks’ capital adequacy ratios increase. When there is a risk of asset depreciation, the proportion of credit losses in banks decreases while the proportion of investment losses increases. Bank bankruptcy risk does not significantly affect the proportion of losses in different financial markets of the multi-layer financial network system.

According to the analysis, for the same size of risk shock, an increase in bank capital adequacy ratio leads to a decreased probability of bank failure due to direct losses, and the spread of risks within the multi-layer financial network system can be more readily interrupted. Therefore, the risk spillover through banks also decreases, and overall, the systemic risk of the multi-layer financial network system has been reduced. Considering that the proportion of firm credit to total bank assets is much higher than the proportion of bank investment and interbank lending, improving capital adequacy is also the most obvious way to mitigate the systemic risk caused by firm credit default. The destructive power of systemic risk on financial markets makes “strong regulation” of the financial sector necessary. As financial agents become increasingly interconnected in the financial market, their decision-making should not only consider the risks of individuals, but also the risks of the entire financial system. Financial agents should be subject to certain regulatory requirements and constraints. This paper argues that financial regulators and banks should hold high regard for the capital adequacy ratios of banks, which are important indicators of their resilience to risk.

**Figure 8:**
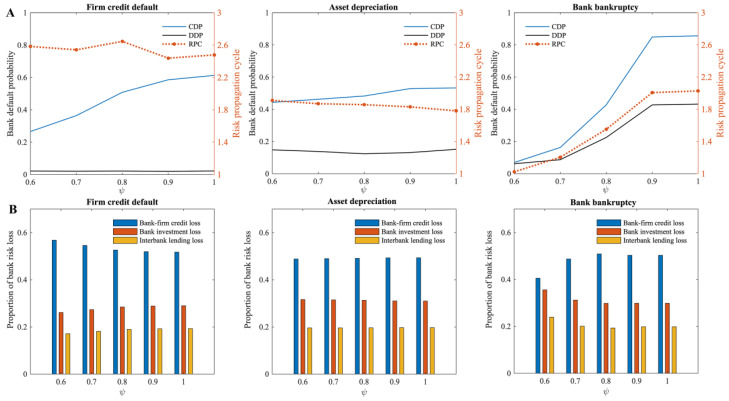
Systemic risk of the multi-layer financial network system under different ψ. ((**A**): The default probability of banks and the risk propagation cycle under different ψ; (**B**): The proportion of bank risk losses in different financial markets under different ψ). CDP indicates the cumulative default probability of banks; DDP indicates the direct default probability of banks; RPC indicates the risk propagation cycle.

In this model, a firm will default when its loan acquisition rate (ψ) is below the minimum loan acquisition rate ($\psi_{0}$) (i.e., the critical value of a firm’s credit default). As shown in Figure 8A, the higher the minimum loan acquisition rate of the firm when the risk occurs, the greater the cumulative default probability of banks in the multi-layer financial network system. According to the risk propagation cycle, when there is a risk of bank bankruptcy, the increase in the minimum loan acquisition rate of the firm prolongs the risk propagation cycle in the multi-layer financial network system. However, when firm credit default risk and asset depreciation risk occur, the minimum loan acquisition rate of the firm has little impact on the cycle of risk propagation. In terms of the proportion of losses in different financial markets (Figure 8B), when firm credit default risk occurs, an increase in the minimum loan acquisition rate of the firm leads to a decrease in the proportion of bank credit losses. The minimum loan acquisition rate of the firm increases the proportion of bank credit losses in total losses when bank bankruptcy risk occurs. However, when asset depreciation risk occurs, the minimum loan acquisition rate of the firm does not have a significant effect on the proportion of losses.

According to the analysis, a higher minimum loan acquisition rate of the firm reflects greater reliance on credit and a lower vulnerability to risk for firms. Based on the left and middle subplots of Figure 8A, when the risk of firm credit default or asset depreciation occurs, the change in the minimum loan acquisition rate of the firm does not significantly change the direct shock to banks, which implies that the risk of spreading outward through banks remains unchanged. As the minimum loan acquisition rate of the firm increases, the firm’s sensitivity to credit losses increases, and the resulting firm credit defaults will continue to rise, which in turn will cause more bank failures, and the cumulative default probability of all banks within the multi-layer financial network system will increase as well. Similarly, bank bankruptcy risk causes firms to suffer credit losses directly, which in turn increases the default probability of firms, leading to a wider range of bank failures through the bank–firm credit linkages. Meanwhile, as the minimum loan acquisition rate of the firm increases, the number of firms that default due to credit losses also increases, intensifying the spread of risks and, in turn, prolonging the risk propagation cycle (the subplot on the right of Figure 8A).

In terms of the proportion of loss from risk (Figure 8B), the number of banks that fail due to direct shocks does not change significantly when the risk of firm credit default occurs. As the minimum loan acquisition rate of the firm increases, there will be an increase in firm credit defaults, which will lead to more bank failures. As a result, outside the bank–firm credit market, losses in other financial markets will also increase, thereby reducing the proportion of bank credit losses in the total losses. When bank bankruptcy risk occurs, the increase in the minimum loan acquisition rate of the firm directly increases firm credit defaults caused by failed banks, which consequently increases the proportion of bank credit losses in total losses. In summary, a higher minimum loan acquisition rate of the firm indicates a stronger reliance of the firm on credit, which will increase the systemic risk of the multi-layer financial network system. To avoid increasing systemic risk, banks should thoroughly evaluate the degree of credit dependence of each firm when providing credit support. On the other hand, to avoid potential risks arising from excessive financial leverage and over-reliance on credit, firms must also pay attention to their indebtedness.

### 3.4. Asset Allocation Strategy

It is noted, in this paper, that banks hold three types of risky assets, and that, when interbank lending assets are considered as a supplement to interbank liquidity, their proportion is generally stable to total assets. In the model presented in this paper, the proportion of interbank lending assets to total assets is set to 20%, and the impact of the banks’ credit assets and portfolio size on the systemic risk of the multi-layer financial network system is mainly examined based on the different asset allocation strategies of the banks. As described in this model, θ denotes the ratio of credit assets held by banks to total assets and η denotes the ratio of portfolio assets held by banks to total assets (according to the settings of this model, θ+η=0.8). Lastly, by adjusting the ratio of credit assets held by banks to total assets, the impact of changes in asset allocation is examined.

**Figure 9:**
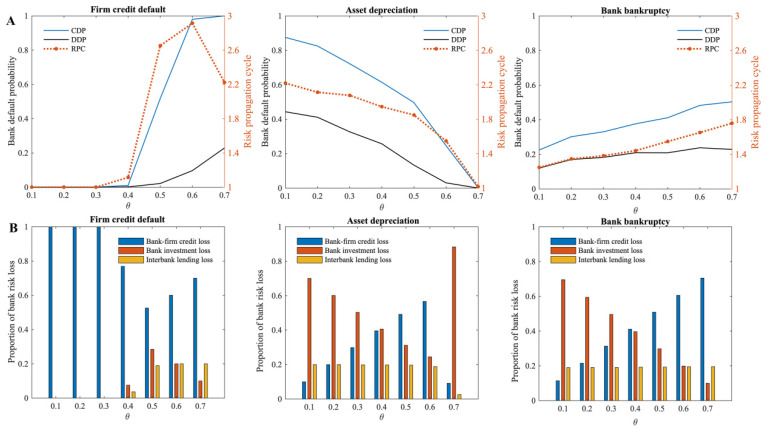
Systemic risk of the multi-layer financial network system under different θ. ((**A**): The default probability of banks and the risk propagation cycle under different θ; (**B**): The proportion of bank risk losses in different financial markets under different θ). CDP indicates the cumulative default probability of banks; DDP indicates the direct default probability of banks; RPC indicates the risk propagation cycle.

According to Figure 9A, when the firm credit default risk or bank bankruptcy risk occurs, the cumulative default probability of banks caused by these risks increases as the ratio (θ) of credit assets held by banks to total assets increases (the left and right subplots of Figure 9A). It is similar to the trend described in Lux’s [34] study. With the increasing proportion of bank credit assets, it is easy to understand that if there is a firm credit default risk, the default risk will spread throughout the system via multiple contagion channels, resulting in increasing bank failures. When it comes to firm credit default risk, the risk propagation cycle reaches a certain point and then falls again, while when it comes to bank bankruptcy risk, the risk propagation cycle increases as the ratio of credit assets held by banks rises. However, when it comes to asset depreciation risk (the subplot in the middle of Figure 9A), the larger the proportion of credit assets held by banks to total assets, the lower the cumulative default probability of banks it causes, and the shorter the risk propagation cycle. In terms of the proportion of risk losses (Figure 9B), the ratio of bank credit losses to total losses decreases when the risk of firm credit default occurs, and then increases as the ratio of credit assets held by banks to total assets increases. Similarly, in the case of asset depreciation risk, the ratio of investment loss to total loss of banks also decreases and then increases. In the case of bank bankruptcy risk, as the proportion of bank credit assets to total assets increases, the credit losses of banks increase, and investment losses decrease.

According to the analysis, the greater the proportion of credit assets held by banks to total assets, the higher the proportion of credit assets held by banks, and the larger the credit risk exposure between banks and firms. When firm credit default occurs, as the proportion of credit assets held by banks increases, the direct shocks suffered by banks continue to rise, and their risk of spillover to other financial markets keeps rising, the higher the cumulative default probability of banks in the whole multi-layer financial network system, and the longer the risk propagation cycle. However, when the direct shocks to banks are too large, more banks fail under the direct shocks, so there is a complete collapse of the banking system in a very short cycle, and the risk propagation cycle decreases instead. Similarly, when bank bankruptcy risk occurs, the greater the ratio of credit assets held by banks to total assets, the greater the credit losses that firms are exposed to and, therefore, the greater the probability of credit default, which indirectly amplifies the shocks faced by the banking system, and the longer the risk propagation cycle. In contrast, the higher the proportion of total assets held by banks in credit assets, the lower the proportion of portfolio assets held by banks. Therefore, when asset depreciation occurs, the smaller its impact on banks, the lower the cumulative default probability of banks in the whole multi-layer financial network system; as the risk loss becomes smaller, the risk propagation cycle becomes shorter.

On the other hand, when the risk of firm credit default occurs, if the ratio of credit assets held by banks to total assets is low (θ≤0.3), the risk loss it causes is small, and it is absorbed by banks directly and cannot be spread to other markets (the subplot on the left of Figure 9B). As the ratio of credit assets held by banks to total assets increases, the credit loss increases, and it begins to spread to other financial markets as a result. The risk loss in this multi-layer financial network system is once again concentrated in the credit market when the ratio of credit assets held by banks to total assets is large (θ>0.5). Therefore, the proportion of bank credit losses in the financial market tends to decrease and then increase under this risk source. As was mentioned above, the greater the proportion of credit assets held by banks to total assets, the smaller the proportion of portfolio assets held by banks. Therefore, when asset depreciation occurs, the bank’s investment losses also show a similar trend. When bank bankruptcy risk occurs, the risk will be transmitted to other banks through the common borrowers and assets held in the multi-layer financial network system. Consequently, the risk losses of banks in the multi-layer financial network system are first concentrated in the portfolio market, and then gradually transferred to the credit market as the market size changes owing to an increase in the proportion of credit assets held by banks to their total assets.

In general, when a bank’s total assets remain constant within the multi-layer financial system, the greater the proportion of total assets held in a single financial market, the more sensitive the bank is to the risk of that market, and the higher the total risk of the banking system. According to the risk–return equivalence principle, the higher the return of an investment, the greater its corresponding risk and uncertainty. This problem is effectively solved by investment diversification theory. It is possible to reduce risk without reducing returns by diversifying investments. Therefore, banks should comprehensively consider the returns and risks in each financial market, pay attention to the proportion of each type of asset to total assets, allocate assets reasonably based on the principle of diversification, avoid excessive concentration of risk in a single financial market, and maximize the return on investment.

## 4. Conclusions

In this paper, multiple agents of banks, firms, and assets are considered, and a model of the multi-layer financial network system is constructed based on bank–firm credit linkages, interbank lending linkages, and bank-asset portfolio linkages. We first examine the systemic risk of the multi-layer financial network system under the shocks of three risk sources, namely, firm credit default, asset depreciation, and bank bankruptcy.

The study found that under the impact of different risk sources, risks are propagated through the multi-layer financial network system with banks as a medium. The greater the risk shock, the higher the cumulative default probability of banks. Furthermore, the greater the risk shock, the greater the risk spills outward through banks, leading to an increasingly long risk propagation cycle at the beginning. It should be noted that when the risk shock is too large, the risk is mainly concentrated in the direct transmission channel and indirect failures triggered by linkages between agents are reduced, therefore, the risk propagation cycle gradually decreases. Moreover, the multi-layer financial network system has different sensitivities to different risk sources. Bank bankruptcy risk will spread to multiple financial markets through bank–firm credit linkages, investment portfolios held by banks, and interbank lending linkages, therefore, the multi-layer financial network system is most sensitive to bank bankruptcy risk, followed by firm credit default risk, and finally asset depreciation risk. In general, different types of banks play different roles under different risk sources, which may be both risk-blocking and risk propagation.

The paper then examines the impact of the multi-layer financial network structure, default risk thresholds, and banks’ asset allocation strategy on the systemic risk of the multi-layer financial network system. According to the research, it is found that when there is a risk of bank bankruptcy or firm credit default, the greater the average degree of the firm in the bank–firm credit network, the more creditor banks of the firm, the more the bank–firm credit risk is dispersed under the risk shock, and the less the risk spills over to other financial markets, helping to reduce the systemic risk and the risk propagation cycle within the multi-layer financial network system. Additionally, under these two risk sources, the greater the minimum loan acquisition rate of firm, the stronger the firm’s reliance on bank credit, and the easier the risk spread through bank–firm credit linkages and interbank lending linkages, resulting in higher systemic risks. When asset depreciation risk occurs, the greater the density of the bank-asset portfolio network, the more diversified the asset losses, and the lower the investment risk of the bank. The increase in the density of the bank-asset portfolio network significantly mitigates the systemic risk under this shock risk. When providing credit to firms, banks should take into consideration the firm’s credit, operations, debt diversification, and credit dependence, and can also appropriately increase the types of the bank’s own investment assets to reduce investment risks.

Furthermore, the study found that under the three risk sources, the greater the bank’s capital adequacy ratio, the lower the direct cumulative default probability of banks, the less risk spillover and, thus, the lower the systemic risk and the shorter risk propagation cycle of the multi-layer financial network system, especially when the firm credit default risk occurs, the improvement effect is most significant. The greater the proportion of credit assets held by banks to total assets, the greater the credit risk exposure between banks and firms when firm credit default risk or bank bankruptcy risk occurs. Increases in the proportion of credit assets held by banks to total assets will result in higher credit losses between banks and firms, further aggravating risk propagation through bank–firm and interbank lending relationships and, therefore, increasing systemic risk. When asset depreciation occurs, however, as the proportion of credit assets held by banks increases, the proportion of asset portfolios held by banks decreases, and its impact on the banking system decreases. Therefore, banks need to be prudent about changes in capital adequacy ratios, respond promptly to changes in asset status, and comprehensively examine the proportion of various asset types to total assets to appropriately allocate assets and avoid excessive risk concentration.

While this paper constructs a multi-layer financial network system model with multiple agents and multiple linkages, it does not yet consider the connections between firms and the portfolio connections between firms and assets. It has also been unable to explore in-depth the transmission paths of financial risks through the above interconnections among different agents in the complex financial network system, as well as obtain the characteristics of systemic risks under various contagion channels. Additionally, the model can be improved by taking into account the returns of assets within the multi-layer financial network. This will be further investigated in future work.

## Figures and Tables

**Table 1:** The balance sheet of the bank.

Assets	Liabilities
Bank–firm credit	Net equity
Investment portfolios	Deposits
Interbank loans	Interbank borrowings
Cash	

**Table 2:** List of key variables and parameters and their measurements.

Symbol	Description
$N_{b},\;N_{f},\;N_{a}$	The number of banks, firms, and types of assets in the multi-layer financial network system
$\lambda_{b},\;\lambda_{f}$	The average connection degree of banks and firms in the bank–firm credit network
$\lambda_{a}$	The average connection degree of each type of asset in the bank-asset portfolio network
θ, η	The average ratio of loans and asset portfolios held by banks to their total assets
ρ	The density of the bank-asset portfolio network
$D_{j}$	The borrowing size of firm j
$NW_{m}$	The total value of asset m
$\psi_{j}(t),\;\psi_{0}$	The loan acquisition rate of firm j at time t and the minimum loan acquisition rate of the firm
$\omega_{i}(t),\;\omega_{0}$	The capital adequacy ratio of bank i at time t and the initial capital adequacy ratio of bank
$V_{ij}^{bank-firm},V_{im}^{bank-asset},V_{ik}^{bank-bank}$	The loans to firm j, the value of asset m and interbank loans to bank k from bank i
$A_{i}^{bank-firm},A_{i}^{bank-asset},A_{i}^{bank-bank},A_{i}^{cash},A_{i}$	The loans to firms, asset portfolios, interbank loans to other banks, cash and the total assets of bank i
$L_{i}^{bank-equity},L_{i}^{bank-deposit},L_{i}^{bank-bank}$	The net equity, depositor savings and interbank borrowing from other banks of bank i

**Table 3:** List of parameter values.

Parameters	Initial Value	Range
$N_{b},\;N_{f},\;N_{a}$	50, 4000, 20	
$\lambda_{b}$	160	
$\lambda_{f}$	2	[1, 5]
θ	50%	[10%, 70%]
η	30%	[10%, 70%]
ρ	0.3	[0.1, 0.5]
$\omega_{0}$	0.1	[0.08, 0.12]
$\psi_{0}$	0.8	[0.6, 1]

## Data Availability

The data used to support the findings of this study are available from the author upon request.
